# The development of a reliable and valid instrument to measure the osteoporosis-related knowledge: validation of the Hungarian version of Osteoporosis Knowledge Assessment Tool (OKAT)

**DOI:** 10.1186/s12889-020-09565-w

**Published:** 2021-04-23

**Authors:** Peter Tardi, Brigitta Szilagyi, Alexandra Makai, Monika Gyuro, Pongrac Acs, Melinda Jaromi, Balint Molics, Marta Hock

**Affiliations:** 1 University of Pécs, Faculty of Health Sciences, Institute of Physiotherapy and Sport Science, Pécs, Hungary; 2 University of Pécs, Faculty of Health Sciences, Doctoral School of Health Sciences, Pécs, Hungary; 3 University of Pécs, Faculty of Health Sciences, Institute of Health Insurance, Pécs, Hungary

**Keywords:** Osteoporosis-related knowledge, Public health, Health education, Patient education, Medical education

## Abstract

**Background:**

Osteoporosis is one of the most common chronic musculoskeletal diseases. Osteoporosis-related knowledge is an important contributor in to prevent osteoporosis. There is no validated reliable questionnaire to measure the knowledge in Hungary. The aim of the study was to validate the Osteoporosis Knowledge Assessment Tool (OKAT) Hungarian version.

**Methods:**

The research was a randomized validation study of a new Hungarian language instrument. The questionnaire was administered to 557 randomly selected healthy women (age between 25 and 44 years) from December 2018 to July 2019 in Baranya county, Hungary. The reliability was examined by the Flesch reading ease and McNemar’s test. We examined item discrimination and item-total correlations, inter-item consistency (Cronbach’s alpha coefficient) and principal component factor analysis.

**Results:**

Significant differences (*p* <  0.001) were reported between total scores and the age categories. Significant (*p* <  0.001) correlation (*r* = 0.25) was found between the education level and the knowledge. Significantly (*p* <  0.001) higher knowledge were found in health care profession (14.53 ± 3.58) than the non-health care profession (9.99 ± 4.04). Participants with osteoporosis or fracture in family history had better knowledge (*p* <  0.001). Flesch reading ease was 44, the questionnaire had a Ferguson’s sigma of 0.94 and a Cronbach’s alpha of 0.81. There were no negative inter-item correlations psychometric properties of the OKAT, all items had more than 70% of correlations (*p* <  0.001).

**Conclusions:**

The Hungarian version of the Osteoporosis Knowledge Assessment Tool is a reliable and objective questionnaire to measure women’s knowledge in Hungary.

## Background

Osteoporosis is a disease affecting the structure of the skeleton as a result low bone mineral density and micro- architectural deterioration of bone tissue is generated. Due to the bone mineral density reduction the risk of fragility increases and because of the muscle mass and muscle strength deterioration the risk of falls also increases which results in higher risk of fractures [1]. It is widely accepted hat this disease is associated with impairments of quality of life following fracture, and increased mortality [2–5].

Nowadays it is becoming a major public health problem particularly in postmenopausal women as the incidence of this disease is getting higher and higher. Worldwide more than 200 million people live with osteoporosis that causes 8.9 million fractures per year [6, 7]. In the United States of America 10.2 million adults were diagnosed with osteoporosis and 43.4 million adults with low bone mass. In terms of gender 8.2 million women and 2.0 million men were diagnosed with osteoporosis and 27.3 million women and 16.1 million men had low bone mass. In Europe the problem is also significant, 22 million women and 5.5 million men were estimated to have osteoporosis and 3.5 million new fragility fractures were sustain per year. In Europe the economic burden of the diagnoses and the treatment of the prior fragility fractures was about € 37 billion [6, 8]. In Hungary 10% of the total population is suffering from osteoporosis [9].

Osteoporosis is a progressive musculoskeletal disease, not curable, but can be slowed down, that is why prevention could be the best therapeutic option [10]. Nowadays the patient education is one of the most important part of the prevention. There is evidence suggesting that osteoporosis knowledge is one contributor to osteoporosis preventive behaviour [11–14]. The most important preventive strategies are increasing the level of physical activity at all ages, cessation of smoking, reduction of alcohol consumption, adequate dietary calcium and vitamin D intake [15–19]. Several studies have reported different levels of osteoporosis knowledge in random, population-based samples and these cross-sectional studies have found relation between levels of osteoporosis knowledge and osteoporosis preventive behaviours [20–22]. Prospective studies have been conflicting with some studies demonstrating increases in osteoporosis knowledge and concurrent improvements in osteoporosis preventive behaviours [23–27]. Various tools are available to assess women’s knowledge about osteoporosis like Facts on Osteoporosis Quiz (FOOQ), the Osteoporosis Questionnaire (OPQ), The Osteoporosis Knowledge Test (OKT) and the Osteoporosis Knowledge Assessment Tool (OKAT). Measurement of osteoporosis knowledge is itself problematic and this may be one of the reasons for the variation in the results of the studies described previously. Regardless of the instrument used, serious lack of knowledge was demonstrated on osteoporosis and its related risk factors [28, 29].

Nothing is known about Hungarian women’s health beliefs and their preventative behaviours regarding osteoporosis, because there were no studies about that and there are no valid and reliable tools to measure osteoporosis-related knowledge and beliefs for Hungarian-speaking women. Determining their knowledge, beliefs and behaviours about osteoporosis could be helpful in developing effective education interventions and guiding public health programs for osteoporosis prevention. We specifically would like to have an instrument in Hungarian to measure the knowledge about osteoporosis in that age when the disease is preventable and with healthy lifestyle the knowledge can protect them against osteoporosis in later life.

The Osteoporosis Knowledge Assessment Tool (OKAT) was developed by Winzenberg et al. [30] and validated to Arabic language by Sayed-Hassan and Bashour [10] to measure the knowledge in women between 25 and 44 year age ranges.

Therefore, the aim of this study was to describe the validation of a method in Hungarian to measure the osteoporosis knowledge in a population-based random sample of 25–44-year-old women.

## Materials and methods

### Validation process

The study was carried out in two phases in Pécs, Baranya County, South-western part of Hungary. In the first phase we have made the translation and adaptation processes of the Hungarian form of the OKAT through 5 stages. The translation process from English to Hungarian was based on the process of forward and backward translation. First the forward translation was made by two independent bilingual translators whose native language is Hungarian after the synthesis of the translations was created by us. In the third stage the backward translation and comparison between the Hungarian and English version was completed by two bilingual translators, one of them was a professional medical translator and the other one was an expert of osteoporosis with high advanced English language certification. The fourth stage included an expert committee discussion. Any discrepancy was reviewed by the committee before a final consensus was reached. In the last stage of the translation and adaptation process of the Hungarian version of the instrument was piloted on thirty women to assure comprehension and ease of administration. Few minor changes were made after the pilot testing that has made it easier for everyone to understand. The final Hungarian version of OKAT went for the second phase of this study, which included an assessment of the test-retest among a convenience sample of 40 women from September 2018 to October 2018 to examine the reliability of the questionnaire. The participants completed the questionnaire twice with 3 weeks apart. The measurement of the osteoporosis-related knowledge was assessed at baseline from December 2018 to July 2019.

### Selection and description of participants

The total population of Hungary in 2019 was 9,772,756 in the last population census, of which 5,096,935 were women. According to the Hungarian Central Statistical Office the female population was 199,944 in the region (Baranya County) of which, 49,051 were women aged between 25 and 44 years of age [31]. Participants from the region were randomly selected in this age range from the University of Pécs (students and workers) with the Unified Online Registration System, from hospitals (healthcare workers) with the Integrated Clinical Center’s nominal roll and from the gyms and fitness rooms (health young women) according to the pass serial numbers.

The main inclusion criteria were apparently healthy women whose native language is Hungarian and who approved to participate in the study. Women were excluded if they had previously had measurement of bone density, thyroid disease, renal failure, malignancy or rheumatoid arthritis, a history of hysterectomy, were on hormone replacement therapy, were pregnant or planning pregnancy within 2 years of study entry or were lactating. Only those women completed the questionnaire anonymously who matched the inclusion criteria, there were no exclusion factors and gave their verbal and written consent after being informed about the aim of the research.

Accordingly, we contacted 600 women, 43 of them did not agree to participate in our examination because of the reasons like “lack of time”; “there is no osteoporosis in my family”; “I am on holiday now”. Based on these reasons our sample included 557 premenopausal women between 25 and 44 years from the region with different levels of education and variant professions. The data collection was executed by paper-based and online self-completed method.

### Technical information

The OKAT is a questionnaire with 20 items with “true”, “false” and “don’t know “options. Each item is coded 0 if an incorrect answer or a “don’t know” answer is given and 1 if the correct answer was given with a total potential score of twenty. This 20-item questionnaire focuses on four basic domains: (1) understanding (symptoms and risk of fracture) of osteoporosis (2) the knowledge of risk factors for osteoporosis (3) knowledge of preventive factors as physical activity and diet relating to osteoporosis and (4) treatment availability [30].

Ethics approval was obtained from the Regional Research Ethics Committee of University of Pécs (registration number was 7498–PTE2018). All participants gave written informed consent to participate in the study.

### Statistics

Data were entered into Microsoft Excel and all statistical analyses were performed using SPSS (Version 24). Descriptive analysis was carried out. The data were expressed by mean ± SD and frequency (%).

The validity and reliability of the osteoporosis knowledge questionnaire were examined by: The Flesch reading ease, where we tested the readability of the OKAT, the scores range from 0 to 100 and value more than 60 means people can readily understand the document. The index of difficulty was calculated by number of correct responses/total number of responses. Value higher than 0.75 means that question was frequently answered correctly. McNemar’s test was used to compare the answers of the OKAT between the test and re-test; thus, to identify non-consistent items. Item discrimination (D-value), which test examine how the items discriminate between patients who have low or high knowledge scores. Item-total correlations, the correlation of an item with the remainder of the scale where the coefficient is < 0.20 was considered poor correlation. We tested the inter-item consistency with Cronbach’s alpha coefficient, where values more than 0.7 was considered acceptable.

Furthermore, principal components factor analysis was extracted the factor of OKAT. Using factor analysis for questionnaires with dichotomous items is problematic, our analysis based on prevision studies (OKAT VAL). The sampling adequacy for the analysis was confirmed by Kaiser–Meyer–Olkin test (KMO = 0.87) and Bartlett’s test of Sphericity (χ2 = 1710.88; *p* <  0.001). EFA was performed through principal component analysis on 20 items and yielded one factors with eigenvalues higher than 1. As the criteria for factor extraction, we applied the factor loading cut off value of 0.30.

## Results

A total of 557 women were recruited who agreed to participate in the study. The average age of participants was 34.45 ± 6.92 years, and there was a wide spread of participants across educational levels history of smoking and alcohol intake. No missing values to any item were reported for the questions (Table 1).

**Table 1:** Women’s characteristics of the sample (*n* = 557) and the total scores of OKAT examination by variables

Age	No. of the cases (n)	Percentage (%)	Mean ± SD	***P*** value
25–29 years	188	33.75	11.85 ± 4.32	< 0.001
30–34 years	90	16.16	11.18 ± 4.15	
35–39 years	90	16.16	11.33 ± 4.33	
40–44 years	189	33.93	10.03 ± 4.06	
**Marital Status**				
Single / Divorced / Widow	131	23.52	10.87 ± 4.56	0.162
In relationship	221	39.68	11.45 ± 4.18	
Married	205	36.8	10.7 ± 4.27	
**Education level**				
Primary education	9	1.62	5.22 ± 3.42	< 0.001
Secondary school (medical)	34	6.09	11.82 ± 3.19	
Secondary school (not medical)	60	10.77	7.68 ± 3.84	
High school	75	13.46	9.61 ± 3.91	
University (Bs)	225	40.39	11.88 ± 3.81	
University (Ms)	140	25.13	11.74 ± 4.38	
University (PhD)	14	2.51	14.43 ± 4.39	
**Professions**				
Health care profession	181	32.49	14.53 ± 3.58	< 0.001
Non-health care profession	376	67.50	9.99 ± 4.04	
**Family history about osteoporosis**				
With osteoporosis	270	48.47	12.08 ± 4.17	< 0.001
Without osteoporosis	287	51.53	10.06 ± 4.13	
**Family history of fracture**				
With fracture	136	24.42	13.4 ± 3.96	< 0.001
Without fracture	343	61.58	10.64 ± 4.04	
Do not know	78	14.00	8.67 ± 3.94	
**History of smoking**				
Do not smoke	430	77.19	11.32 ± 4.04	= 0.024
Average 1 to 5 cigarette per day	37	6.64	10.76 ± 4.36	
Average 6 to 10 cigarette per day	45	8.08	10.4 ± 5.19	
Average 10 to 20 cigarette per day	36	6.46	8.75 ± 4.78	
On average more than 1 box per day	9	1.62	11.33 ± 4.95	
**Alcohol intake**				
Never	83	14.90	11.19 ± 4.58	= 0.152
Occasionally (1–2 times a year)	246	44.17	11.19 ± 4.04	
Every 3 month	117	21.01	11.19 ± 4.14	
Several times a month	75	13.46	10.76 ± 4.56	
Weekly	30	5.39	10.47 ± 4.56	
Several times a week	6	1.08	6.17 ± 2.79	
**Health promotion (prevention)**				
I do lot for it	55	9.87	12.6 ± 4.73	< 0.001
I do several things for it	242	43.44	11.61 ± 4.23	
I do few for it	221	39.68	10.49 ± 3.93	
I do very few for it	30	5.39	9.27 ± 4.1	
I do not care for it	9	1.68	5.66 ± 3.28	
**Health status**				
Excellent	75	13.46	13.05 ± 4.25	< 0.001
Very good	255	45.78	11.1 ± 4.03	
Good	201	36.09	10.53 ± 4.14	
Fair	23	4.13	9 ± 5.75	
Poor	3	0.54	7.75 ± 3.5	

Significant differences (*p* <  0.001) were reported between total scores of OKAT and the age categories. In younger age the knowledge was higher, and it decreased with aging. There were significant differences (*p* <  0.001) in the education levels and between the types of the professions. It was observed that with higher level of education combined with higher level of osteoporosis-related knowledge. The results were higher in the healthcare professions. Higher osteoporosis-related knowledge was found with osteoporosis and fracture by osteoporosis in the family history furthermore with better health status and promotion the knowledge about osteoporosis was significantly higher (Table 1.)

Significant (*p* <  0.001) correlation was found between the education level (*r* = 0.25) and the osteoporosis-related knowledge furthermore with the health status (*r* = 0.18) and negative significant (*p* <  0.001) correlation (*r* = − 0.18) with the ages. Based on these with higher level of general education women have higher knowledge about osteoporosis as well as with better knowledge participants have more appropriate health status, over and above appreciable that the osteoporosis-related knowledge is getting worse with aging.

The English version of the OKAT had a Flesch reading ease of 45, the Hungarian form had almost the same with 44. If the word osteoporosis were removed from the English version questions the reading ease rose to 65, while the Hungarian form rose to 53.

The index of difficulty for most items was satisfactory (between 0.25 and 0.716). Items 1, 4, 8 and 15 scored above 0.75, indicating that most participants answered correctly.

The percentage of correct answers as shown is based on Winzenberg et al. [30], furthermore on the latest European guidance for the diagnosis and management of osteoporosis in postmenopausal women according to Kanis et al. [2] (Table 2.)

**Table 2:** Correct answers for the OKAT among the study sample and at test/re-test sample (*n* = 40)

Correct answers (%)							McNemar’s tests (***p*** value)
	All (***n*** = 557)		Test (***n*** = 40)		Retest (***n*** = 40)		
	N	%	N	%	N	%	
**1. Osteoporosis leads to an increased risk of bone fractures.**	541	95.92	38	95.00	38	97.44	1.000
**2. Osteoporosis usually causes symptoms (e.g. pain) before fractures occur.**	307	54.43	17	42.50	18	46.15	1.000
**3. Having a higher peak bone mass at the end of childhood gives no protection against the development of osteoporosis in later life.**	179	31.85	15	37.50	17	43.59	0.375
**4. Osteoporosis is more common in men.**	451	79.96	31	77.50	30	76.92	1.000
**5. Cigarette smoking can contribute to osteoporosis.**	389	68.97	24	60.00	26	66.67	0.250
**6. White women are at highest risk of fracture as compared to other races.**	271	48.05	24	60.00	23	58.97	1.000
**7. A fall is just as important as low bone strength in causing fractures.**	357	63.41	30	75.00	28	71.79	1.000
**8. By age 80, the majority of women have osteoporosis.**	451	79.96	33	82.50	32	82.05	1.000
**9. From age 50, most women can expect at least one fracture before they die.**	250	44.33	18	45.00	17	43.59	1.000
**10. Any type of physical activity is beneficial for osteoporosis.**	141	25.00	12	30.00	11	28.21	1.000
**11. It is easy to tell whether I am at risk of osteoporosis by my clinical risk factors.**	283	50.18	24	60.00	24	61.54	1.000
**12. Family history of osteoporosis strongly predisposes a person to osteoporosis.**	404	71.63	32	80.00	31	79.49	1.000
**13. An adequate calcium intake can be achieved from two glasses of milk a day.**	312	55.32	23	57.50	22	56.41	1.000
**14. Sardines and broccoli are good sources of calcium for people who cannot take dairy products.**	350	62.17	27	67.50	28	71.79	0.500
**15. Calcium supplements alone can prevent bone loss.**	426	75.53	32	80.00	31	79.49	1.000
**16. Alcohol in moderation has little effect on osteoporosis.**	221	39.18	17	42.50	17	43.59	1.000
**17. A high salt intake is a risk factor for osteoporosis.**	160	28.47	14	35.00	16	41.03	0.500
**18. There is a small amount of bone loss in the ten years following the onset of menopause.**	304	54.00	14	35.00	15	38.46	1.000
**19. Hormone therapy prevents further bone loss at any age after menopause.**	131	23.27	8	20.00	6	15.38	1.000
**20. There are no effective treatments for osteoporosis available in “Hungary”**^**a**^**.**	296	52.48	21	52.50	20	51.28	1.000

^a^ Originally Australia

The reliability statistics for all the 20 items of the Hungarian version of the OKAT was high, Cronbach’s alpha was 0.81 which is excellent, and it is in the acceptability range (0.7–0.85).

The mean D-value (item discrimination) for the questionnaire was 50.4%. Discriminatory power was measured by Ferguson’s sigma which was 0.94 for the questionnaire.

According to the item-total correlations psychometric properties of the OKAT, there were no negative inter-item correlations. All items had more than 70% of correlations which were significant (*p* <  0.001) and so were satisfactory (Table 3.)

**Table 3:** Psychometric Properties of the OKAT by Item

Item Number	Item Discrimination (%)	Item-total correlation	***P*** value	Factor Loading
**1. Osteoporosis leads to an increased risk of bone fractures.**	12.42	0,7	< 0.001	0.61
**2. Osteoporosis usually causes symptoms (e.g. pain) before fractures occur.**	58.33	0.85	< 0.001	0.47
**3. Having a higher peak bone mass at the end of childhood gives no protection against the development of osteoporosis in later life.**	78.42	1.00	< 0.001	0.45
**4. Osteoporosis is more common in men.**	46.35	0.94	< 0.001	0.56
**5. Cigarette smoking can contribute to osteoporosis.**	44.95	0.83	< 0.001	0.43
**6. White women are at highest risk of fracture as compared to other races.**	66.67	1.00	< 0.001	0.43
**7. A fall is just as important as low bone strength in causing fractures.**	34.71	1.00	< 0.001	0.72
**8. By age 80, the majority of women have osteoporosis.**	51.61	0.84	< 0.001	0.47
**9. From age 50, most women can expect at least one fracture before they die.**	45.05	0.89	< 0.001	0.52
**10. Any type of physical activity is beneficial for osteoporosis.**	62.38	0.95	< 0.001	0.48
**11. It is easy to tell whether I am at risk of osteoporosis by my clinical risk factors.**	41.76	0.89	< 0.001	0.48
**12. Family history of osteoporosis strongly predisposes a person to osteoporosis.**	41.47	0.91	< 0.001	0.45
**13. An adequate calcium intake can be achieved from two glasses of milk a day.**	53.69	0.85	< 0.001	0.66
**14. Sardines and broccoli are good sources of calcium for people who cannot take dairy products.**	58.99	0.74	< 0.001	0.39
**15. Calcium supplements alone can prevent bone loss.**	50.54	1.00	< 0.001	0.38
**16. Alcohol in moderation has little effect on osteoporosis.**	39.04	0.87	< 0.001	0.71
**17. A high salt intake is a risk factor for osteoporosis.**	42.22	1.00	< 0.001	0.56
**18. There is a small amount of bone loss in the ten years following the onset of menopause.**	47.65	1.00	< 0.001	0.42
**19. Hormone therapy prevents further bone loss at any age after menopause.**	70.12	0.95	< 0.001	0.49
**20. There are no effective treatments for osteoporosis available in “Hungary”**^**a**^**.**	62.33	0.95	< 0.001	0.45

^a^ Originally Australia

## Discussion

This study aimed to validate and test the reliability of the Hungarian version of the OKAT, which is the first questionnaire in Hungarian that measures the knowledge about osteoporosis. The questionnaire is developed for premenopausal healthy women between 25 and 44 years. We have made the data collection with 557 women in this age group. No missing values to any item were reported for the questions. The OKAT performed satisfactorily on virtually all components of the analysis.

Cross sectional studies have reported that the levels of osteoporosis knowledge in random, population-based samples are higher in younger ages and higher level of education also defines it [12, 15, 32]. This difference is visible in our study too. We also have found significant difference between total scores of OKAT and the age categories. The osteoporosis-related knowledge was higher with younger age. Significant differences were also found in the knowledge score between the education levels and profession types. The higher the education level was, the greater the knowledge about osteoporosis was confirmed. Several studies have showed that healthcare students and workers osteoporosis-related knowledge is better than non-healthcare workers [10, 12, 33]. In the comparison of the professions the healthcare workers had significantly better knowledge compared to the non-healthcare workers in our study too.

The questionnaire had a Flesch reading ease of 44 which is similar to the original (45) English version [30]. It was due to the word osteoporosis; it is in 12 of the 20 items. If this word was removed the Flesch reading ease became more acceptable. The word osteoporosis was the most accurate description of the disease about which knowledge was being measured and it is widely recognised in the general population.

The index of difficulty for most items was satisfactory because almost every item value was between the optimal 0.25 and 0.716. Items 1 *(Osteoporosis leads to an increased risk of bone fractures.)*, item 4 *(Osteoporosis is more common in men.)*, item 8 *(By age 80, the majority of women have osteoporosis.)* and item 15 *(Calcium supplements alone can prevent bone loss.)* scored above 0.75. This fact indicating that these four questions were easy to answer and most of the participants answered them correctly.

The mean D-value, that measures the item discrimination for the questionnaire was 50.4%. Discriminatory power for the questionnaire was measured by Ferguson’s sigma what was close to the ideal value of 1.0 with the value 0.94. Though factor analysis must be interpreted cautiously when analysing dichotomous variables, the fact that the analysis did not generate factor with an eigenvalue above 1 is consistent with osteoporosis knowledge being the main factor being measured by the questionnaire. This provides some support for the construct validity of the instrument.

The Hungarian version of OKAT showed consistent findings between the test and retest results. The Cronbach’s alpha of the original English version was 0.69, which was satisfactory for the authors. In our study the Hungarian version got excellent 0.81 that is in the acceptability range (0.7–0.85). The OKAT must include very easy items as well as very difficult items, which tends to decrease the internal consistency of the scale Cronbach’s alpha.

In developed countries, including the Central European countries, like Hungary the prevalence and incidence of osteoporosis is significantly high due to the technical development and the increasing expected lifetime. The prevention should take bigger role in increasing the osteoporosis-related knowledge in order to maintain the osteoporosis preventive behaviours namely raising the level of the physical activity and the intake of adequate calcium and vitamin D in the ages when the disease can be preventable [34].

The Hungarian version of the OKAT is the first questionnaire that can measures the knowledge in young Hungarian-speaking women. The Hungarian version of the OKAT covers the core of osteoporosis-related knowledge; it can be useful in further follow up studies to measure the effectiveness of the preventable education programs.

### Limitations

However, the results confirmed that OKAT is a valid and reliable tool to examine the Hungarian population’s osteoporosis-related knowledge, the study has a number of potential limitations. The sample was randomly selected, but selection bias is possible due to the moderate response rate. By ages, we have created four categories (25–29 years; 30–34 years; 35–39 years; 40–44 years). In the first category (33.75%) and in the fourth (33.93%) the number of the participants was significantly higher, than in the other two categories that could also influence the results. However, our sample was quite big there were only few women with basic primary education, while the number of the participants with Bs and Ms. Degree were quite high.

## Conclusion

This study’s aim was to validate the OKAT Hungarian form in a randomly selected sample. Based on the results we achieved the Hungarian version of the OKAT is a reliable and objective method to measure women’s knowledge in Hungary between 25 and 44 years. Further research is needed with a representative sample to measure the osteoporosis-related knowledge with this tool to get normal values for the Hungarian population.

## Data Availability

All data generated or analysed during this study are included in this published article [and its supplementary information files].
